# Variation, Modification and Engineering of Lipid A in Endotoxin of Gram-Negative Bacteria

**DOI:** 10.3390/ijms22052281

**Published:** 2021-02-25

**Authors:** Kazuyoshi Kawahara

**Affiliations:** Department of Biosciences, College of Science and Engineering, Kanto Gakuin University, Yokohama, Kanagawa 236-8501, Japan; kawahara@kanto-gakuin.ac.jp

**Keywords:** lipopolysaccharide, lipid A, endotoxin, chemical structure, fatty acid, engineering

## Abstract

Lipid A of Gram-negative bacteria is known to represent a central role for the immunological activity of endotoxin. Chemical structure and biosynthetic pathways as well as specific receptors on phagocytic cells had been clarified by the beginning of the 21st century. Although the lipid A of enterobacteria including *Escherichia coli* share a common structure, other Gram-negative bacteria belonging to various classes of the phylum *Proteobacteria* and other taxonomical groups show wide variety of lipid A structure with relatively decreased endotoxic activity compared to that of *E. coli*. The structural diversity is produced from the difference of chain length of 3-hydroxy fatty acids and non-hydroxy fatty acids linked to their hydroxyl groups. In some bacteria, glucosamine in the backbone is substituted by another amino sugar, or phosphate groups bound to the backbone are modified. The variation of structure is also introduced by the enzymes that can modify electrostatic charges or acylation profiles of lipid A during or after its synthesis. Furthermore, lipid A structure can be artificially modified or engineered by the disruption and introduction of biosynthetic genes especially those of acyltransferases. These technologies may produce novel vaccine adjuvants or antagonistic drugs derived from endotoxin in the future.

## 1. Introduction

**Figure 1:**
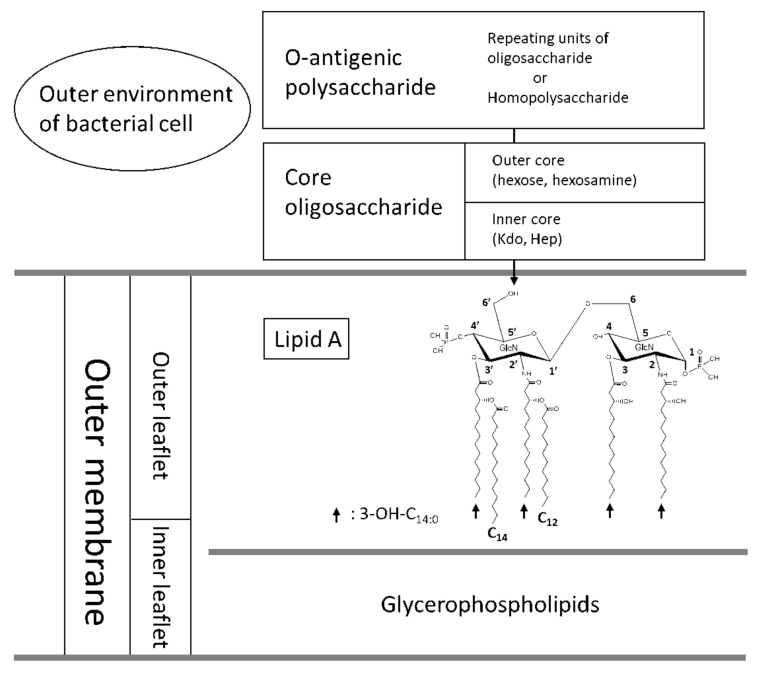
Schematic elucidation of outer membrane and chemical structure of lipid A region with the numbering of carbon atoms in the glucosamine–disaccharide backbone.

Although the term “endotoxin” implies a toxin produced by bacteria, it has another biochemical name, “lipopolysaccharide”. Both terms properly suggest its immunological activities and function as a cell surface component of most Gram-negative bacteria that protect bacterial cells from a harsh outside environment. The lipopolysaccharide (LPS) of enterobacteria including *Escherichia coli*, and that of many other bacteria, consists of three distinct portions. The O-antigenic polysaccharide is an antigenic polymer with wide variety of structure, and the core-oligosaccharide is composed of hexoses, hexosamines, L-*glycero*-D-*manno*-heptose, and 3-deoxy-D-*manno*-octulosonic acid (Kdo) that links the core and lipid A portions. Lipid A works as a lipid anchor to the outer membrane, and represents a central role for the endotoxic activity (Figure 1) [1]. In the present review, the history of endotoxin study, especially the establishment of the chemical structure of lipid A, and the discovery of a receptor specific to lipid A are summarized first. The variation of lipid A structure in relation to bacterial taxonomy is then described, followed by the modification of lipid A structure by genes of its biosynthetic enzymes.

## 2. Confirmation of Lipid A Structure and Discovery of a Lipid A-Specific Receptor

The study of endotoxin was started by R. Pfeiffer at the end of the 19th century [2] during the study of *Vibrio cholerae* pathogenicity by R. Koch. The term “lipopolysaccharide”, abbreviated as LPS, was first used by Shear in the study using *Serratia marcescens* [3]. Despite these early studies, we had to wait for confirmation of the chemical structure of lipid A and its central role in endotoxin activity in the 1980s when the structure of *E. coli*-type lipid A was chemically synthesized by Shiba, Kusumoto, and their colleagues at Osaka University [4,5]. These results were achieved by the close collaboration with Lüderitz, Galanos, Rietschel, and many other German researchers who had elaborated the extraction methods and accumulated experimental data on endotoxin [6,7,8]. At about the same time, the biosynthetic pathways of lipid A and the lipid A-Kdo region were intensively investigated by Raetz and his collaborators in USA [9], and lipid A was chemically and biochemically elucidated. Figure 1 shows the structure of *E. coli* lipid A, which represents the most immunologically active form among lipid A molecules from various Gram-negative bacteria.

Even after lipid A was chemically clarified, receptors of immune cells that recognize lipid A to induce immunological activity were kept mysterious. In 1990, the function of CD14 expressed on the plasma membrane of macrophages was reported, and mechanisms of binding with the aid of an LPS-binding protein (LBP) were investigated [10]. However, the search for the final receptor on phagocytic cells continued, and finally in 1998, just after the Toll receptor of *Drosophila* was discovered [11], the transmembrane leucine-rich protein, Toll-like receptor 4 (TLR4), was identified as a receptor specific to endotoxin (lipid A) [12]. TLR4 functions with a specific protein MD-2 to recognize lipid A, and transduces the binding information through protein kinases to NF-κB and other transcription factors that induce expression of pro-inflammatory cytokine genes such as IL-1β, IL-6, or TNF-α in phagocytic cells [13,14]. We can therefore realize that the history of endotoxin research started in the 19th century, and the platform of knowledge necessary for further applicational study had been established at the beginning of the 21st century.

## 3. Diversity of Lipid A Structure in Various Taxonomic Groups of Gram-Negative Bacteria

Gram-negative bacteria generally have a cell surface structure with cytoplasmic and outer membranes, and most of them, but not all, have LPS with a polar oligosaccharide region and lipid A as an anchor on the outer leaflet of the outer membrane. However, LPSs or lipid As that exhibit strong immunological activity are limited to bacteria of certain taxonomical groups, whereas many others have LPS with little immunological activity compared to that of *E. coli* and taxonomically related bacteria.

**Figure 2:**
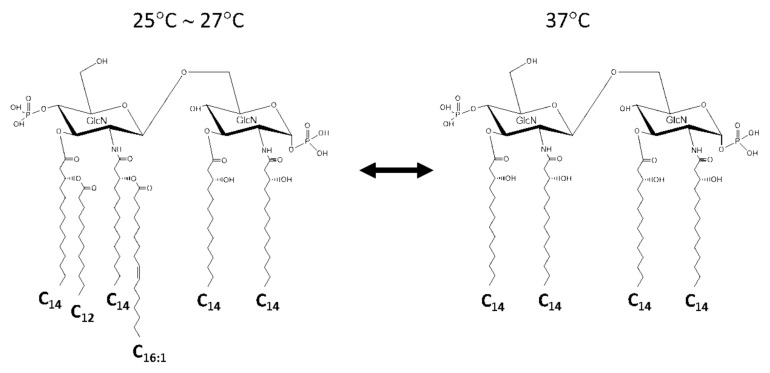
Structural conversion of *Yersinia pestis* lipid A by growth temperature (25 ~ 27 °C: environmental temperature, 37 °C: human body temperature).

It is widely known that the most active lipid A recognized by TLR4 on dendritic cells and macrophages is “*E. coli*-type” lipid A (Figure 1) [1]. Most of the members of the family *Enterobacteriaceae* in the class *γ-Proteobacteria* share a principle lipid A structure consisting of a β1,6-linked glucosamine (GlcN) dimer backbone, phosphate groups at the 1 and 4′ positions of the backbone, and four molecules of 3-hydroxymyristic acid (3-OH-C_14:0_) linked to the 2, 3, 2′, and 3′ positions of the backbone (see Figure 1 for numbering of carbon atoms in the backbone). The non-hydroxy fatty acids esterifying the hydroxyl groups of 3-OH-C_14:0_ linked to the distal (non-reducing) GlcN have, to some extent, diversity among genera. *Klebsiella pneumoniae* produces lipid A with myristic acid (C_14:0_) at 3-OH-C_14:0_ residues linked to the 2′ and 3′ positions in addition to the *E. coli*-type lipid A [15,16]. *Yersinia pestis* and *Y. pseudotuberculosis* substitute the hydroxyl group of 3-OH-C_14:0_ at the 2′ position with palmitoleic acid (C_16:1_) and that at the 3′ position with lauric acid (C_12:0_) at lower growth temperatures (25–27 °C). These fatty acids are nevertheless absent at 37 °C, the body temperature of humans and other mammals (Figure 2) [17,18]. *Yersinia enterocolitica* also shows the temperature-dependent lipid A modification, but the acylation profile is different from the above two species of this genus [18,19]. *Salmonella enterica* strains as well as other members of enterobacteria possess the *pagP* gene, which expresses an acyltransferase that can transfer palmitic acid (C_16:0_) to the hydroxyl group of 3-OH-C_14:0_ at the 2 position of the backbone to produce hepta-acylated lipid A [20]. This conversion of lipid A acylation is believed to confer resistance of the bacterium against bactericidal agents such as defensins and LL-37 in phagocytic cells or the ability to survive in certain harsh environments [21]. Another popular group in *γ-Proteobacteria* is the family *Pseudomonadaceae*. *Pseudomonas aeruginosa* and taxonomically related bacteria contain lipid A with fatty acids of shorter carbon chains [22,23]. The 2 and 2′ positions of the backbone are substituted by 3-hydroxylauric acid (3-OH-C_12:0_), whereas the 3 and 3′ positions are substituted (but not fully) by 3-hydroxycapric acid (3-OH-C_10:0_). The hydroxyl groups of two 3-OH-C_12:0_ residues are acylated by C_12:0_ or 2-hydroxylauric acid (2-OH-C_12:0_). The latter fatty acid is formed by the oxygenation [24], which is described later in Section 4. Species of the genus *Legionella* form a phylogenetic cluster apart from the group containing *Enterobacteriaceae* and *Pseudomonadaceae*. This taxonomic distance is reflected by structural differences of lipid A. *Legionella pneumophila*, a causative agent of lethal pneumonia, has a lipid A with a backbone composed of a 2,3-diamino-2,3-dideoxy-glucose (GlcN3N) disaccharide, amide-linked fatty acids of 14–22 carbon atoms, and long-chain fatty acids such as 27-oxo-fatty acid with 28 carbon atoms ester-linked to iso-C_14:0_ at the 3′ position [25]. The presence of this distinct long-chain fatty acid suggests a relation with the family *Rhizobiaceae* in the class *α-Proteobacteria* as described later in this section.

**Figure 3:**
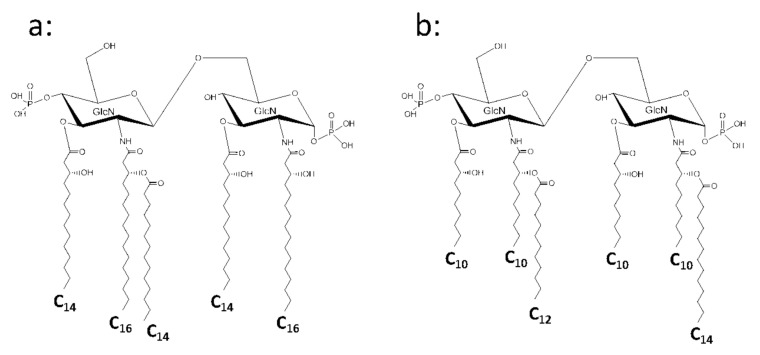
Chemical structure of lipid A from *Burkholderia cepacia* (**a**) and *Comamonas testosteroni* (**b**).

The chain length of 3-hydroxy-fatty acids also shows a wide variety among species in the class *β-Proteobacteria*. The plant pathogen *Burkholderia cepacia* has a lipid A with 3-hydroxypalmitic acid (3-OH-C_16:0_) at the 2 and 2′ positions, and one of them at the 2′ position is substituted by C_14:0_. The 3 and 3′ positions are substituted by the common 3-OH-C_14:0_ (Figure 3a) [26,27]. The causative agent of melioidosis, *B. pseudomallei*, has a similar substitution pattern of fatty acids in its lipid A [28,29]. *Bordetella pertussis*, a causative agent of whooping cough, is also a member of this class. The lipid A of this pathogen and related bacteria has been intensively studied by several groups including that of Caroff et al. in France [30,31,32]. This lipid A contains 3-OH-C_10:0_ at the 3 position of the backbone, and the 2, 2′, and 3′ positions are substituted by 3-OH-C_14:0_, one of which bound at the 2′ position is substituted by C_14:0_, representing a rather asymmetric lipid A structure. Modification of this lipid A is described in Section 5. Another member of *β-Proteobacteria*, *Comamonas testosteroni*, a resident of soil and water environments, has a lipid A of a symmetric structure with only a shorter 3-hydroxy fatty acid, 3-OH-C_10:0_, at all of the 2, 2′, 3, and 3′ positions, and the hydroxyl groups of those at the 2 and 2′ positions are substituted by C_14:0_ and C_12:0_, respectively (Figure 3b) [33]. *Neisseria meningitidis* also has a symmetric lipid A with 3-OH-C_12:0_ at the 3 and 3′ positions and 3-OH-C_14:0_ esterified with C_12:0_ at the 2 and 2′ positions [34].

**Figure 4:**
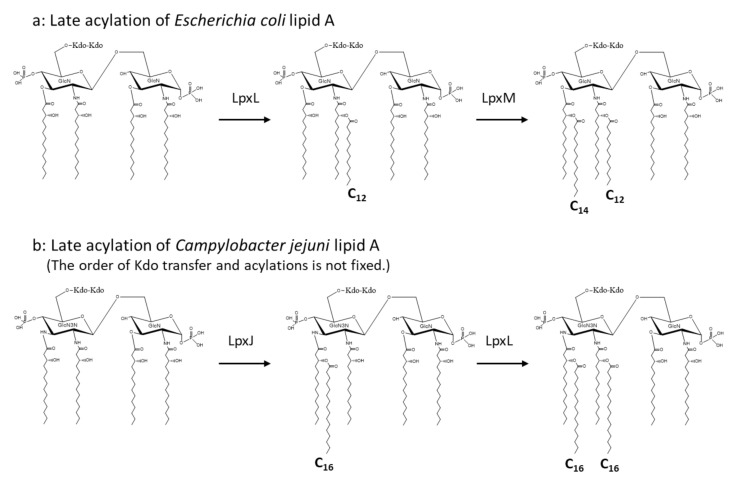
Acylation to form acyloxyacyl structure at the late stage of lipid A biosynthesis of *Escherichia coli* (**a**) and *Campylobacter jejuni* (**b**).

*Helicobacter pylori* is a member of the class *ε-Proteobacteria*, and a known cause of chronic gastritis. Its lipid A is composed of a reduced number of fatty acids [35,36]. It contains 3-hydroxy fatty acids with 16 and 18 carbon atoms, one of which at the 2′ position is substituted by stearic acid (C_18:0_). The 1 position of proximal (reducing end) GlcN is substituted by phosphate as other lipid A molecules, but the 4′ position of distal GlcN is unsubstituted. The predominant lipid A molecule is tetraacylated, but Suda et al. reported an even less acylated lipid A of this pathogen [37]. *Campylobacter jejuni* is also a member of *ε-Proteobacteria*, and its lipid A has a backbone composed of GlcN3N and GlcN as distal and proximal sugars, respectively. Although the sugar backbone is different from that of a usual lipid A, the chain lengths of 3-hydroxy fatty acids at the 2, 2′, 3, and 3′ positions are identical to those of *E. coli*, and two 3-OH-C_14:0_ molecules at the distal sugar are ester linked by C_16:0_ (Figure 4b) [38,39]. It should be noted that the transfer of C_16:0_ was reported to occur first to 3-OH-C_14:0_ bound to the 3′ position of GlcN3N by the transferase LpxJ, followed by the second transfer of C_16:0_ to 3-OH-C_14:0_ of the 2′ position by another transferase LpxL (Figure 4b). This order of acylation is different from that of *E. coli* (Figure 4a), but is commonly found among the genera *Helicobacter* and *Campylobacter* [40].

**Figure 5:**
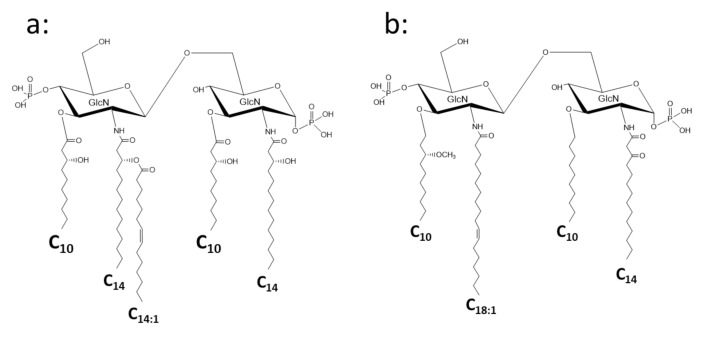
Chemical structure of *Rhodobacter sphaeroides* lipid A (**a**) and Eritoran (**b**) developed as an antagonist of endotoxin based on the structure of *R. sphaeroides* lipid A.

**Figure 6:**
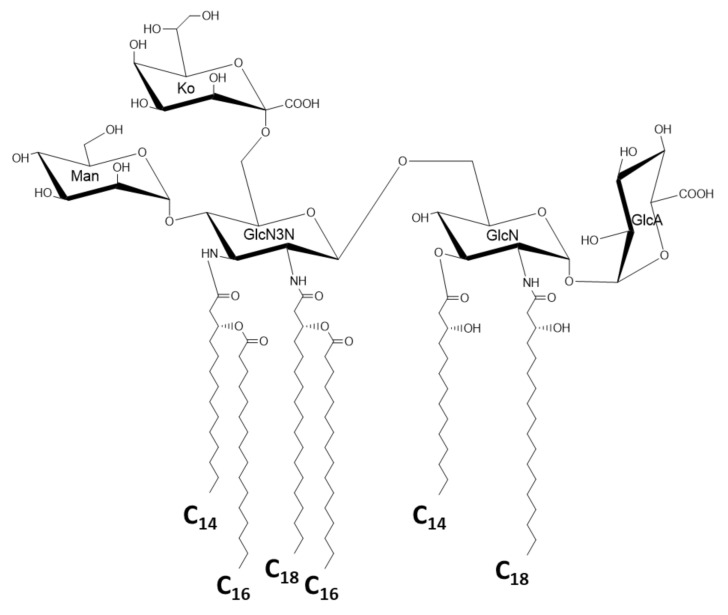
Chemical structure of *Acetobacter pasteurianus* lipid A with the sugar component (Ko) of the core oligosaccharide at the 6′ position of the backbone.

A group of bacteria that shows great structural variety of lipid A in the phylum *Proteobacteria* is the class *α-Proteobacteria*. A photosynthetic bacterium, *Rhodobacter sphaeroides* has a lipid A with 3-OH-C_14:0_ at the 2 and 2′ positions and 3-OH-C_10:0_ at the 3 and 3′ positions, and 3-OH-C_14:0_ at the 2′ position is substituted by tetradecenoic acid (C_14:1_) (Figure 5a) [41,42]. This lipid A is the model for the lipid A antagonist Eritoran, which was developed by Eisai Co. as a drug for severe sepsis (Figure 5b) [43,44]. Lipid A of the nodule-forming and N_2_-fixing bacterium, *Rhizobium legminosarum*, lacks phosphate groups at both the 1 and 4′ positions of the backbone, and galacturonic acid substitutes the 4′ position to supply a negative charge to this lipid A. It contains 27-hydroxyoctacosanoic acid (27-OH-C_28:0_) with a 3-hydroxybutyrate ester linked to the 27-OH-group [45,46]. A similar lipid A structure with very long fatty acids and hopanoid was also found in *Bradyrhizobium* strains [47]. *Acetobacter* species, acetate-forming bacteria that are popular in the food industry, belong to *α-Proteobacteria* as well. The lipid A of *A. pasteurianus* was recently investigated, and some features common with those of *Rhizobium* and *Bradyrhizobium* species were elucidated (Figure 6) [48,49]. This lipid A lacks phosphate, and the backbone is substituted by glucuronic acid and mannose at the 1 and 4′ positions, respectively. The backbone contains GlcN3N as a distal sugar, which was also found in *Bradyrhizobium* lipid A. Interestingly, the lipid A of *A. pasteurianus* links to the core oligosaccharide through D-*glycero*-D-*talo*-octulosonic acid (Ko) [48], which was first found in *Acinetobacter* LPS [50], and then in other species [51,52,53]. This sugar is deduced to stabilize the acid–labile linkage between lipid A and the core to adjust to acidic growth conditions. The class *α-Proteobacteria* also contains obligate intracellular parasites, *Rickettsia* species. Unexpectedly, the lipid A of *Rickettsia typhi* conserves the basic structure similar to that of *E. coli*, although the fatty acids are longer [54,55]. It contains 3-OH-C_16:0_ as amide linked fatty acids at the 2 and 2′ positions, 3-OH-C_14:0_ as ester linked fatty acids at the 3 and 3′ positions, and C_16:0_ linked to 3-hydroxy fatty acids at the distal GlcN. Among many unique taxonomic groups in the class *α-Proteobacteria*, the families *Sphingomonadaceae* and *Erythrobacteriaceae* need to be described here, because members of these families are known to lack LPS, and instead, glycosphingolipids are present [56,57]. These glycosphingolipids contain glucuronic acid or galacturonic acid linked to ceramide, and exhibit immunostimulatory activity through activation of natural killer T-cells [58].

The phylum *Bacteroidetes* is independent from the large phylum *Proteobacteria*, but the basic structure of lipid A is conserved by most of the members of this phylum. The lipid A of *Bacteroides fragilis* has a sugar backbone identical to that of the family *Enterobacteriaceae*, and is similarly acylated, although the chain length and molecular species of fatty acids are different. The 2′ position is substituted by 3-hydroxy iso-heptadecanoic acid (3-OH-iso-C_17:0_) with its hydroxyl group esterified with iso-pentadecanoic acid (iso-C_15:0_). The 2, 3, and 3′ positions are substituted by 3-OH-C_16:0_, 3-hydroxypentadecanoic acid (3-OH-C_15:0_), and 3-OH-C_16:0_, respectively, whereas the 4′ position is not phosphorylated [59]. Another member of this phylum is the family *Porphyromonadaceae*. *Prophyromonas gingivalis* is a causative agent of periodontal disease, but recently thought to be involved in many other diseases [60]. The lipid A is similar to that of *B. fragilis*. It partially lacks the phosphate group at the 4′ position, and is acylated by 3-OH-iso-C_17:0_ at the 2 and 2′ positions. One of 3-OH-iso-C_17:0_ bound to the 2′ position is substituted by C_16:0_. The 3 and 3′ positions are substituted by 3-OH-C_16:0_ and 3-hydroxy iso-pentadecanoic acid (3-OH-iso-C_15:0_), respectively [61,62]. Recently, one species of this phylum, *Aureispira marina*, isolated from marine debris [63], was found to contain only trace amounts of 3-hydroxy fatty acids and a large amount of ceramide, suggesting a lack of LPS in this bacterium [64].

It is not easy to summarize the correlation between lipid A structure and bacterial taxonomy as the diversity of the structure is very wide, and the structural difference does not always correspond to the taxonomic distance, as we see the similarity of lipid A of the families *Bacteroidaceae* and *Enterobacteriacea* that belong to the different phylums. Nevertheless, informative tables presenting bacteria–lipid A structure relationships are found in some review articles [65,66].

## 4. Structural Modification of Lipid A after the Synthesis of LPS

After lipid A−core region is synthesized at the inner surface of the cytoplasmic membrane, it is transported to the outer surface of the membrane [67]. At this stage, the phosphate groups at the 1 and 4′ positions of the backbone may be modified by positively charged hydrophilic molecules. Colistin-resistant strains of *E. coli* and other enteric bacteria have lipid A with phosphoethanolamine bound to one of the phosphate groups in the lipid A molecule [68,69]. The plasmid-encoded gene *mcr-1* and its variant genes are responsible for the transferases. Additionally, aminoarabinose transferase can transfer 4-aminoarabinose (Ara4N) to the phosphate groups [70]. These enzymes are widely distributed among species of *Enterobacteriaceae* and also other families of *Proteobacteria* [71,72]. Phosphoethanolamine and Ara4N can neutralize the negative charges of the phosphate groups and disturb the action of cationic antibiotics or antibacterial agents. However, these substitutions do not directly affect the recognition of lipid A by TLR4 and MD-2 [73].

On the contrary, modifications of the fatty acid profile greatly influence the binding of lipid A to TLR4/MD-2, and they can occur even after mature LPS is transported to the outer membrane. C_16:0_ transferase, known as PagP in *Salmonella* species, resides in the outer membrane, and transfers C_16:0_ to the hydroxyl group of 3-OH-C_14:0_, which is linked to the amino group of the 2 position, to form hepta-acylated lipid A [21]. Enzymes homologous to PagP are widely present among species of the family *Enterobacteriaceae* and other species of *Proteobacteria* [74]. *Salmonella* species also have a modifying enzyme known as PagL, which liberates 3-OH-C_14:0_ from the 3 position of lipid A [75]. Homologous enzymes were found in a variety of species in *Proteobacteria* [76]. Another deacylating enzyme LpxR is present in enterohaemorrhagic *E. coli*, *Salmonella*, and several other bacteria [77,78]. The enzyme can liberate 3-OH-C_14:0_ at the 3′ position together with the non-hydroxy fatty acids bound to it. These acylating and deacylating modifications certainly affect the hydrophobicity, the conformation, and the affinity between lipid A and its receptor TLR4, and reduce the immunoactivity of lipid A or the lipid A-containing LPS [79,80]. By these modifications described above, lipid A can change the electrostatic charge and hydrophobicity, and also the rigidity and fluidity of the outer membrane to adjust bacterial cells to the living environment or that of infection.

Different from modification by acylation or deacylation, *Salmonella enterica* has an enzyme that hydroxylates C_14:0_ at the distal GlcN of lipid A to 2-hydroxymyristic acid (2-OH-C_14:0_) [81]. This hydroxylation of fatty acid in lipid A was suggested to be an oxygenation reaction using ^18^O_2_ in an early study of *Pseudomonas* lipid A [82], and genetically proved by using *Salmonella* [81,83]. The substrate of this hydroxylation was assumed to be fully acylated Kdo-Kdo-lipid A, indicating that this enzyme is also one of those that can work after the synthesis of mature LPS. 2-Hydroxy fatty acids are major components of lipid A molecules from aerobic species such as *Pseudomonas*, *Chromobacterium*, *Acinetobacter*, *Bordetella*, and many other genera [84]. In the case of *Pseudomonas aeruginosa* and *Acinetobacter baumannii*, the hydroxylation was reported to have some relation with the pathogenicity and infectivity [24,85], but no influence on the membrane integrity. However, the physiological meaning of this hydroxylation should be further investigated if we consider the presence of 2-hydroxy fatty acids in many non-pathogenic bacteria.

Most of lipid A-modifying enzymes described in this section are known to be regulated by the two component systems, PhoP–PhoQ and PmrA–PmrB [86]. The environmental conditions such as pH and concentration of divalent cations or nutrition influence the lipid A structure and also the survival of bacteria through these two-component systems.

## 5. Construction of Novel Lipid A Structure by Genetic Engineering

Until the early 1980s, little was known about the biosynthetic pathways of lipid A. Codeveloped with structural studies, all of the synthetic pathways had been elucidated in a few decades, and the genes for the enzymes involved were cloned [87,88]. With the accumulation of this knowledge, we now have a possibility to create a novel lipid A structures with a desirable immunological activity. However, simple cloning or manipulation of genes does not always produce a novel structure, because each enzyme has substrate specificity, and foreign intermediates may not be recognized as substrates for the next reaction, resulting in very low yield of product. Nevertheless, several successful examples are found in recent studies.

Li et al. [89] constructed mutant strains of *E. coli* with different acylation patterns using the *pagL* gene and previously reported mutant strains of acyltransferases [90,91]. Needham et al. [92] generated library of strains by the combination of two *E. coli* mutant strains and genes of phosphatases from *Francisella tularensis* and the acyl chain-modifying enzymes described above in this review. They demonstrated that variety of lipid A molecules with graded TLR4-dependent cytokine responses can be produced.

Bainbridge et al. [93] constructed an *E. coli* strain defective of the *lpxL* gene and harboring a cloned *Bacteroides* C_16:0_ transferase gene. The recombinant *E. coli* strain produced lipid A with C_16:0_ instead of C_12:0_ as the acyl chain bound to 3-OH-C_14:0_ at the 2′ position. This strain was used to investigate the effects of intestinal microbiota for inflammatory bowel disease [94].

Our group also constructed an *E. coli* mutant that lacks *lpxL* and the *pagP*-homologous gene (*crcA*), and used it for lipid A modification [95]. The mutant strain still has *lpxM* (C_14:0_ transferase gene), but transfers little amount of C_14:0_ to 3-OH-C_14:0_ at the 3′ position. With the plasmid-encoded *pagP* from *Salmonella*, the *E. coli* strain produced the lipid A with only one acyloxyacyl structure with C_16:0_ bound to 3-OH-C_14:0_ at the 2 position in proximal GlcN. Interestingly, the IL-6-inducing activity of the LPS from this strain was only slightly recovered, although the growth rate and resistance to polymyxin B became nearly normal. When *lpxL2*, a C_14:0_ transferase gene of *Klebsiella pneumoniae* [96], was introduced instead of *pagP*, the strain produced lipid A with two C_14:0_ molecules bound to 3-OH-C_14:0_ at the 2′ and 3′ positions. The LPS from this strain showed IL-6-inducing activity comparable to that of the wild-type strain [97].

**Figure 7:**
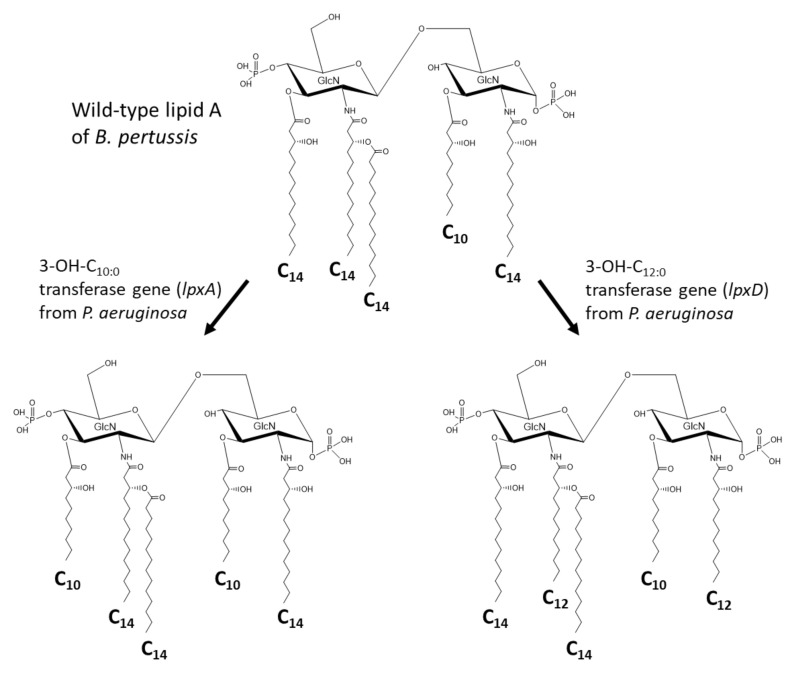
Engineering of *Bordetella pertussis* lipid A using acyltransferase genes from *Pseudomonas aeruginosa*.

Arenas et al. [98] engineered *Bordetella pertussis* to shorten the acyl chain length in the lipid A structure. Introduction of the *lpxA* gene for a 3-OH-C_10:0_ transferase from *P. aeruginosa* changed the asymmetric structure of the molecule to symmetric by the substitution of 3-OH-C_14:0_ with 3-OH-C_10:0_ (Figure 7). Similarly, introduction of the *lpxD* gene for a 3-OH-C_12:0_ transferase from the same bacterium changed the fatty acids at the 2 and 2′ positions to 3-OH-C_12:0_ (Figure 7). The LPS of the recombinant *B. pertussis* strain exhibited lower endotoxic activity, suggesting the possibility of a new whole-cell pertussis vaccine with reduced pyrogenicity. They also succeeded to introduce the *lpxL* gene (C_12:0_ transferase gene) from *Neisseria meningitidis* and the *lpxL* gene (C_16:0_ transferase gene) from *Porphyromonas gingivalis*, and substituted ester-linked C_14:0_ by C_12:0_ and C_16:0_, respectively.

These reported examples demonstrate that artificial modification or engineering of lipid A structure is possible if we utilize appropriate acyltransferase genes, and other genes for lipid A synthesis, if necessary, cloned from foreign bacterial strains. Mutant strains defective of one or more acyltransferase genes and lacking an acyloxyacyl structure in lipid A are useful tools for lipid A engineering. Such mutant strains can be constructed as reported by several groups including ours [91,92,93,95,99], because the acyloxyacyl structure is not essential for the function of lipid A in the outer membrane, although in most cases the mutant strains grow slowly and are susceptible to antibacterial drugs. Additionally, the reports on engineered *B. pertussis* strains [98,100] showed the possibility that fatty acids directly bound to the sugar backbone can also be substituted if we choose the proper combination of acyltransferase genes and host bacteria.

## 6. Closing Remarks

As demonstrated in this review, variations of lipid A in various Gram-negative bacteria are unlimited. Novel lipid A structures will be found and reported further in the future, which would provide us models for the structural modification. Lipid A engineering is attractive because it may produce lipid A molecules with expected or unexpected immunological activity, and may improve lipid A-derived vaccine adjuvants [101,102,103] that are highly required at present for the new generation vaccine against emerging infectious diseases. Moreover, it may help to develop the lipid A-antagonistic drugs such as Eritoran (Figure 5b) to rescue the septic shock caused by bacterial infections. Thus, the diversity of microorganisms and their genes for lipid A biosynthesis will give us the possibility to create new drugs derived from endotoxin.

## Data Availability

Not Applicable.

## Abbreviations

LPS	lipopolysaccharide
Kdo	3-deoxy-D-*manno*-octulosonic acid
TLR4	Toll-like receptor 4
GlcN	glucosamine
GlcN3N	2,3-diamino-2,3-dideoxy-glucose
Ko	D-*glycero*-D-*talo*-octulosonic acid
Ara4N	4-aminoarabinose
C_12:0_	lauric acid
C_14:0_	myristic acid
C_16:0_	palmitic acid
3-OH-C_10:0_	3-hydroxycapric acid
3-OH-C_12:0_	3-hydroxylauric acid
3-OH-C_14:0_	3-hydroxymyristic acid
3-OH-C_16:0_	3-hydroxypalmitic acid
3-OH-iso-C_17:0_	3-hydroxy iso-heptadecanoic acid
